# Nasal administration of anti-CD3 monoclonal antibody ameliorates disease in a mouse model of Alzheimer’s disease

**DOI:** 10.1073/pnas.2309221120

**Published:** 2023-09-05

**Authors:** Juliana R. Lopes, Xiaoming Zhang, Julia Mayrink, Bruna K. Tatematsu, Lydia Guo, Danielle S. LeServe, Hadi Abou-El-Hassan, Felipe Rong, Maria J. Dalton, Marilia G. Oliveira, Toby B. Lanser, Lei Liu, Oleg Butovsky, Rafael M. Rezende, Howard L. Weiner

**Affiliations:** ^a^Ann Romney Center for Neurologic Diseases, Department of Neurology, Brigham and Women’s Hospital, Harvard Medical School, Boston, MA 02115; ^b^The Gene Lay Institute of Immunology and Inflammation, Brigham and Women’s Hospital, Harvard Medical School, Boston, MA 02115

**Keywords:** nasal anti-CD3, 3xTg mice, microglia, T cells

## Abstract

Emerging evidence suggests that dysregulation of neuroinflammation, particularly that orchestrated by microglia, plays a significant role in the pathogenesis of Alzheimer’s disease (AD). Danger signals including dead neurons, dystrophic axons, phosphorylated tau, and amyloid plaques alter the functional phenotype of microglia from a homeostatic (M0) to a neurodegenerative or disease-associated phenotype, which in turn drives neuroinflammation and promotes disease. Thus, therapies that target microglia activation constitute a unique approach for treating AD. Here, we report that nasally administered anti-CD3 monoclonal antibody in the 3xTg AD mouse model reduced microglial activation and improved cognition independent of amyloid beta deposition. In addition, gene expression analysis demonstrated decreased oxidative stress, increased axogenesis and synaptic organization, and metabolic changes in the hippocampus and cortex of nasal anti-CD3 treated animals. The beneficial effect of nasal anti-CD3 was associated with the accumulation of T cells in the brain where they were in close contact with microglial cells. Taken together, our findings identify nasal anti-CD3 as a unique form of immunotherapy to treat Alzheimer’s disease independent of amyloid beta targeting.

## Significance

Alzheimer’s disease (AD) is a neurodegenerative disease characterized by amyloid plaques, neurofibrillary tangles, and microglial activation. Therapies targeting amyloid beta have shown positive effects in subjects with AD. Nasal anti-CD3 has been shown to treat animals with a progressive form of experimental autoimmune encephalomyelitis, a model for multiple sclerosis, by inducing regulatory T cells that dampen microglial inflammation in the brain. Here, we show that nasal anti-CD3 also ameliorates disease in a murine model of AD by targeting microglial activation in the brain independent of amyloid beta deposition. These studies identify a unique approach to treat Alzheimer’s disease that could also be given in combination with antiamyloid therapy.

Alzheimer’s disease (AD) is the most prevalent neurodegenerative disorder in the United States with 6.5 million Americans aged 65 or older with the disease (1). AD is characterized by amyloid beta (Aβ) plaques and neurofibrillary tangles in the brain (2–4). These changes trigger neuroinflammation and neuronal death which in turn is associated with cognitive impairment (5).

Neuroinflammation is a major component of AD (5–9). Studies showing activated microglia and astrocytes surrounding Aβ plaques suggest significant involvement of inflammatory pathways in the disease (6, 7, 10, 11). Although rare forms of dominantly inherited AD are linked to mutations in APP or presenilin genes, the majority of genetic risk factors linked to late-onset Alzheimer’s involve genes that are enriched or expressed in immune cells, especially microglia and macrophages, including APOE, triggering receptor expressed on myeloid cells 2 (TREM2), Adenosine triphosphate (ATP)-binding cassette transporter (ABCA) family, complement, cluster of differentiation 33 (CD33), human leucocyte antigen (HLA)-family, MADS box transcription enhancer factor 2C (MEF2C), and membrane-spanning 4A (MS4A) family (12, 13).

Microglia are the primary immune cells of the brain that help both maintain homeostasis and react to injury. As such they play a central role in central nervous system (CNS) disease processes. It is now recognized that following CNS inflammation, microglia change from a homeostatic phenotype to a neurodegenerative (MGnD) (14) or disease-associated microglia (DAM) (15).

A growing body of evidence has suggested important roles of adaptive immune cells in promoting or inhibiting inflammation associated with AD. Regulatory T cells (Tregs) are key modulators of immune responses, and play a critical role in maintaining immunological tolerance and in suppressing excessive immune responses deleterious to the host (16). Several Treg subtypes have been described including Foxp3^+^ Tregs and Foxp3^−^IL-10^+^ Tregs, also known as Tr1 cells. A beneficial role for Tregs in AD has been reported by several investigators (17–21). Baek et al. showed that adoptive transfer of Tregs into the 3xTg mouse model of AD improved cognition and reduced Aβ deposition, whereas depletion of Tregs aggravated spatial learning deficits in the same mice (19). Consistent with this, in the APP/PS1 mouse model of AD, Dansokho et al. showed that depletion of Tregs accelerated the onset of cognitive deficits triggered by Aβ deposition and that induction of Treg expansion by low doses of IL-2 improved cognitive function in these mice without reducing amyloid plaque burden (17). Importantly, such cognitive improvement was associated with increased microglia recruitment toward Aβ deposition (17). In human studies, Faridar et al. found that subjects with AD had Treg dysfunction, which was restored by ex vivo expansion of Tregs that in turn down-regulated activated macrophages (20). Moreover, Ciccocioppo et al found a significant decrease in Tregs in AD patients (21). Thus, there is clear evidence of Treg dysfunction in AD and that enhancing Treg functions would be expected to be beneficial to the treatment of AD.

In this context, we have found that nasal administration of anti-CD3 monoclonal antibody ameliorated disease in mouse models of multiple sclerosis (MS) (22), diabetes (23), lupus (24), and arthritis (25) by the induction of Tregs. In studies most relevant to the treatment of AD by nasal anti-CD3, we found that nasal anti-CD3 treatment in a progressive model of experimental autoimmune encephalomyelitis (EAE), a mouse model for MS, dampened microglia and astrocyte inflammation in the CNS (22). Importantly, in human subjects, we have found that the nasal administration of a fully human anti-CD3 antibody (Foralumab) modulated immune responses when given to normal subjects (26) and showed positive effects in a pilot trial in subjects with COVID-19 with minimal toxicity (27, 28). Given this and the importance of activated microglia in AD, we tested nasal anti-CD3 administration in the 3xTg mouse model of AD and found modulation of activated microglia, changes in gene expression patterns in the brain, and improved cognition independent of Aβ deposition. Thus, nasal anti-CD3 has the potential to be a nontoxic immunotherapeutic approach for the treatment of AD.

## Results

### Nasal Anti-CD3 Improves Cognition in the 3xTg Mouse Model of AD.

**Fig. 1.**
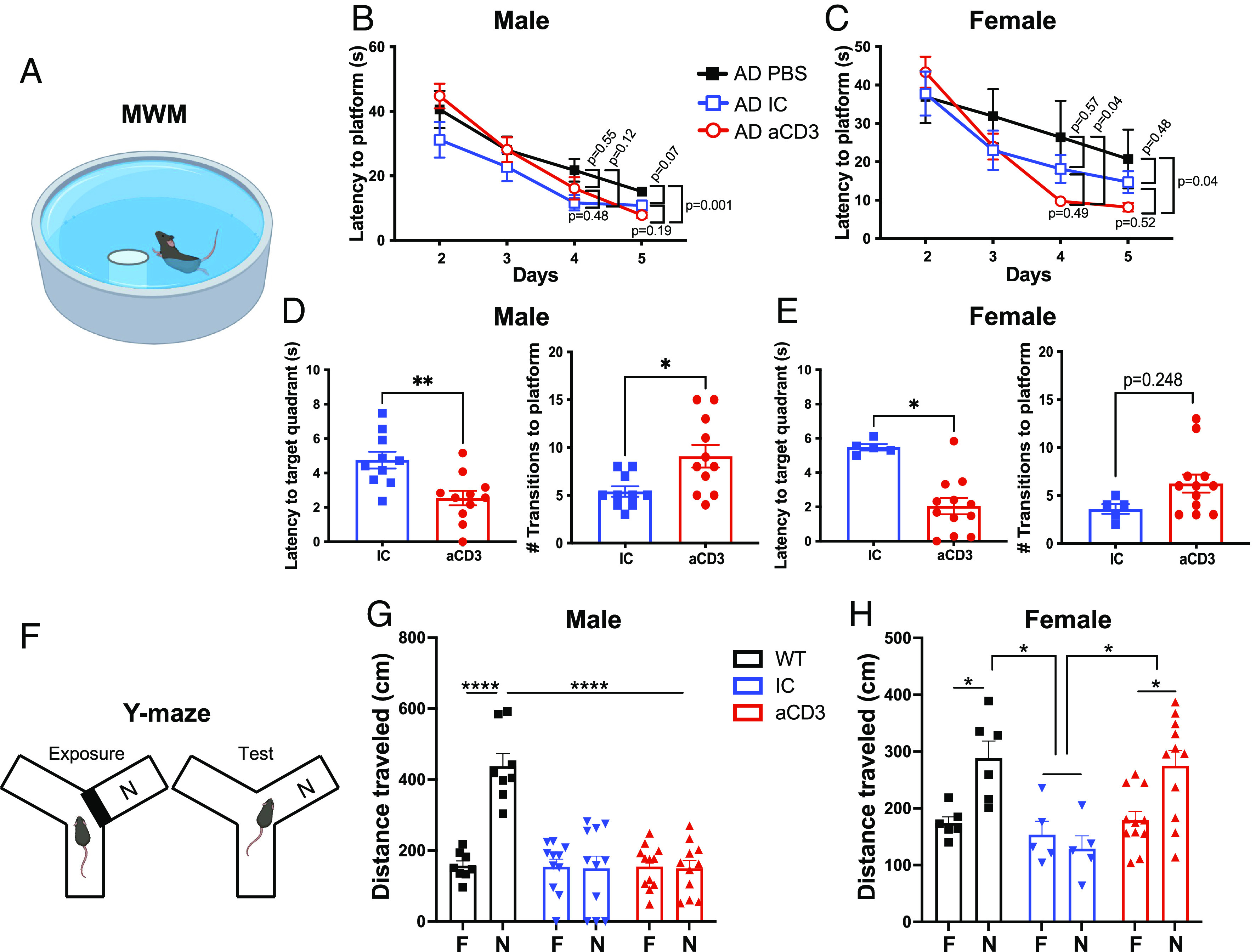
Nasal anti-CD3 ameliorates cognition in 3xTg mice. (*A*) Representative image of the Morris water maze (MWM) test. (*B* and *C*) Latency to platform in sec measured on days 2, 3, 4, and 5 in the MWM test of male (*B*) and female (*C*) mice treated with 1 µg of either nasal anti-CD3 (aCD3), isotype control (IC), or PBS 3×/week for 5 mo starting at 1 mo of age. Males: n = 8 PBS, 10 IC, 11 aCD3. Females: n = 8 PBS, 5 IC, 12 aCD3. (*D* and *E*) Latency to target quadrant in sec and number of transitions to platform on day 6 (probe day) in the MWM test of male (*D*) and female (*E*) treated mice. Males: n = 10 IC, 11 aCD3. Females: n = 5 IC, 12 aCD3. Student’s *t* test. (*F*) Representative image of the novel arm Y-maze test. (*G* and *H*) Distance traveled in cm in the familiar (F) or novel arms (N) of the Y-maze of male (*G*) and female (*H*) treated mice with 1 µg of either nasal anti-CD3 (aCD3) or isotype control (IC) 3×/week for 5 mo starting at 1 mo of age. Wild-type (WT) C57BL/6 mice were used as controls. Male: n = 8 WT, 11 IC, 12 aCD3. Female: n = 6 WT, 5 IC, 11 aCD3. One-way ANOVA followed by Tukey’s posttest for multiple comparisons. Data are mean ± SEM. *P < 0.05, **P < 0.01, ****P < 0.0001.

To investigate whether nasal anti-CD3 affected cognition in the 3xTg mouse model of AD, we tested the effect of nasal anti-CD3 on spatial learning, and long- and short-term memories using the Morris water maze (MWM) and the novel arm Y-maze behavioral tests. One-month-old 3xTg mice were treated with 1 µg/mouse of either nasal anti-CD3, isotype control (IC), or phosphate buffered saline (PBS) three times a week for 5 mo (*SI Appendix*, Fig. S1*A*) then MWM and Y-maze were performed. We found that in the MWM test (Fig. 1*A*), which measures spatial learning and long-term memory, nasal anti-CD3 improved cognition in both male and females as shown by decreased time to reach the platform during training days (day 5 for males and days 4 and 5 for females) (Fig. 1 *B* and *C*) and by decreased time to reach the target quadrant and increased number of transitions to quadrant (Fig. 1 *D* and *E*). Of note, we did not find differences in locomotion between groups as measured by total distance traveled in the MWM test (*SI Appendix*, Fig. S1*B*). In the novel arm Y-maze test (Fig. 1*F*), which measures short-term memory, nasal anti-CD3 improved memory in females, but not males as measured by increased distance traveled in the novel arm (Fig. 1 *G* and *H*).

We then investigated the effect of nasal anti-CD3 on Aβ accumulation. In 3xTg mice, Aβ accumulates intraneuronally (29) and thus we measured intracellular Aβ by immunofluorescence (IF) in the CA1 of hippocampus, prefrontal cortex (PC), and retrosplenial cortex (RSC) (29). We found no differences in intracellular Aβ in either male or female 3xTg mice treated with nasal anti-CD3 (*SI Appendix*, Fig. S1 *C–E*). Since Aβ increases at later stages of the disease in 3xTg mice, we measured soluble Aβ oligomers from brain extracts of 3xTg mice treated with nasal anti-CD3, IC, or PBS for 11 mo (treatment started when mice were at 1 mo of age and they were killed at 12 mo of age) and found no difference between groups and in either male or female mice (*SI Appendix*, Fig. S1*F*).

Taken together, nasal administration of anti-CD3 mAb improves disease in 3xTg mice, with a slight difference between males and females. In addition, improved cognition induced by nasal anti-CD3 in the 3xTg mouse model of AD is not associated with Aβ modulation.

### Nasal Anti-CD3 Modulates Gene Expression in the Cortex and Hippocampus.

Since nasal anti-CD3 improved cognition in 3xTg mice independent of neuronal Aβ accumulation (*SI Appendix*, Fig. S1), we investigated whether nasal anti-CD3 affected gene expression in the cortex and hippocampus, two critical brain areas affected in 3xTg mice (29, 30). We harvested brains from 3xTg mice treated with nasal anti-CD3 as described in *SI Appendix*, Fig. S1*A*, isolated the cortex and hippocampus, and performed gene expression analysis using the Nanostring neuropathology panel. We found that nasal anti-CD3 altered the transcriptional landscape of both the cortex (*SI Appendix*, Fig. S2 and Dataset S1) and hippocampus (*SI Appendix*, Fig. S3 and Dataset S2) in both males and females, though the genes affected were different. In the cortex, pathway analysis of male mice showed that nasal anti-CD3 up-regulated genes associated with axogenesis, synapsis organization, response to amyloid beta, and carbohydrate homeostasis (*SI Appendix*, Fig. S2 *A–C*) and down-regulated genes related to oxidative stress, neuron death, catecholamine secretion, and regulation of neurotransmitter levels (*SI Appendix*, Fig. S2 *D* and *E*). Pathway analysis in female mice showed upregulation of genes associated with calcium transport, autophagy, and neuron projection organization (*SI Appendix*, Fig. S2 *F–H*) and downregulation of genes related to glial cell development, central nervous system myelination, and regulation of neurogenesis (*SI Appendix*, Fig. S2 *I* and *J*).

In the hippocampus of male mice, nasal anti-CD3 up-regulated genes associated with the regulation of metal ion, autophagy, and cysteine-type endopeptidase activity (*SI Appendix*, Fig. S3 *A–C*), whereas it down-regulated genes related to the regulation of peptidyl-tyrosine phosphorylation, nitric oxide biosynthetic, and carbohydrate metabolic processes (*SI Appendix*, Fig. S3 *A*, *D*, and *E*). In the hippocampus of female mice, the majority of differentially expressed genes (DEGs) were down-regulated (30 out of 34 genes; Dataset S2) and pathway analysis of down-regulated genes involved axogenesis, dendritic spine development, and regulation of intracellular transport (*SI Appendix*, Fig. S3 *F*–*H*). Notably, only six genes in the cortex and one gene in the hippocampus were shared between male and female 3xTg mice treated with nasal anti-CD3 or IC (Datasets S1 and S2). Moreover, gene expression analysis of untreated 6-mo-old male vs. female 3xTg mice showed differences (*P* < 0.05) in DEGs between sexes. In the cortex, there were 32 DEGs between males and females in a total of 770 genes (4.15%); and in the hippocampus, there were 31 DEGs between males and females in a total of 770 genes (4%) (Dataset S4). Thus, nasal anti-CD3 modulates genes in the cortex and hippocampus in a sex-dependent manner.

### Nasal Anti-CD3 Modulates Microglial Phenotype.

**Fig. 2.**
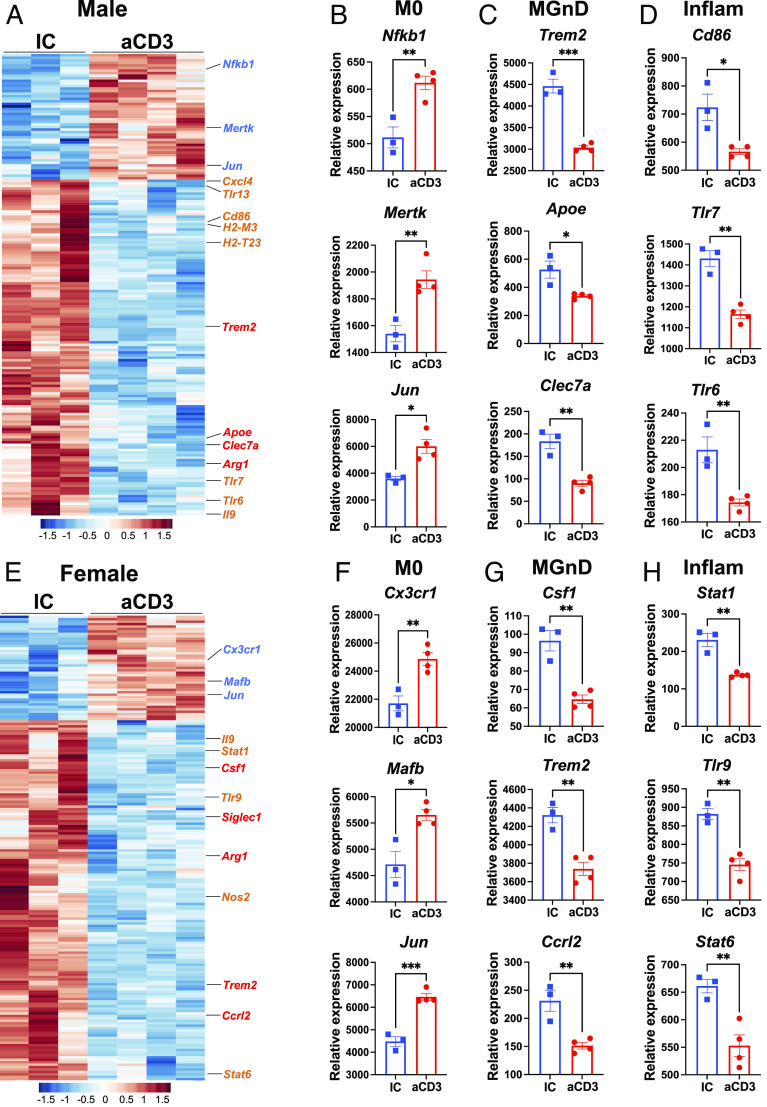
Nasal anti-CD3 reduces microglial activation in 3xTg mice. Microglial cells were sorted after the termination of the 5-mo treatment of male and female mice with 1 µg of either nasal anti-CD3 (aCD3) or isotype control (IC) 3×/week starting at 1 mo of age and gene expression analysis performed using the Nanostring mouse myeloid panel. (*A*–*D*) Heatmap (*A*) and representative genes of homeostatic (M0; *B*), neurodegenerative (MGnD; *C*), and inflammatory (Inflam.; *D*) from male mice. n = 3 IC, 4 aCD3. Student’s *t* test. (*E*–*H*) Heatmap (*E*) and representative genes of M0 (*F*), MGnD (*G*) and Inflam. (*H*) from male mice. n = 3 IC, 4 aCD3. Student’s *t* test. Data are mean ± SEM. *P < 0.05, **P < 0.01, ***P < 0.001.

To further investigate the mechanism by which nasal anti-CD3 ameliorates disease in 3xTg mice, we asked whether nasal anti-CD3 modulated the microglial signature as microglial cells are known to become activated in neurodegenerative disorders including AD (31). We sorted microglia from 3xTg mice treated with nasal anti-CD3 or IC three times a week for 5 mo (see *SI Appendix*, Fig. S1 *A* and *E* for gating strategy) using the microglia-specific marker 4D4 (31, 32) and performed gene expression analysis using the Nanostring mouse myeloid panel, comparing nasal anti-CD3 vs. IC-treated mice. Consistent with our previous findings (31), both male and female untreated animals showed an activated microglial profile characterized by increased expression of MGnD and inflammation-related genes and decreased expression of M0 genes (Fig. 2 and Dataset S3). In males, we found that nasal anti-CD3 induced upregulation of 49 genes and downregulation of 130 genes (*P* < 0.05) compared to IC-treated mice (Dataset S1; Fig. 2 *A–D*). In females, nasal anti-CD3 induced upregulation of 42 genes and downregulation of 147 genes (*P* < 0.05) (Dataset S1 and Fig. 2 *E*–*H*). Notably, 109 (41.6%) of the genes were shared between males and females, 71 (21.1%) were exclusively seen in males, and 82 (31.3%) were exclusively seen in females (Dataset S5). We found that nasal anti-CD3 up-regulated M0 genes including *Nfkb1*, *Mertk*, *Jun*, *Cx3cr1,* and *Mafb* (Fig. 2 *A*–*C*) and down-regulated MGnD genes including *Trem2*, *Apoe*, *Clec7a*, *Csf1*, and *Ccrl2* (Fig. 2 *E*–*G*) in both males and females. Interestingly, nasal anti-CD3 also down-regulated genes associated with inflammation and antigen presentation including *Cd86*, *Tlr7*, *Tlr6*, *Stat1*, *Tlr9*, and *Stat6* in both males and females (Fig. 2 *A*, *C*, *E*, and *H*). Taken together, these findings demonstrate that nasal anti-CD3 modulates the microglial transcriptomic signature in 3xTg mice.

### Nasal Anti-CD3 Expands Regulatory T Cells in the Periphery.

We have previously shown in the EAE model that nasal anti-CD3 acts by inducing IL-10 producing Tregs (22). To investigate whether nasal anti-CD3 induced IL-10+ Tregs in 3xTg mice, we performed flow cytometric analysis of both the spleen and cLN of mice treated three times a week for 5 mo with anti-CD3 or IC. We also treated naive C57BL/6 mice (B6) with PBS as a control for peripheral immune cell changes induced by AD pathology in 3xTg mice. In the spleen, nasal anti-CD3 increased the frequency of CD4+IL10+ Tregs in both males and females vs. IC. (*SI Appendix*, Fig. S4 *A* and *B*). In the cLN, both nasal anti-CD3 and IC-treated mice had increased frequencies of CD4+IL-10+ cells in males but not in females (*SI Appendix*, Fig. S4 *C* and *D*). It is important to note that the increase in %CD4+IL10+ cells in the IC group vs. B6 in males was not related to an increase in %CD4+IL-10+ cells induced by IC but rather to an elevated basal level of these cells in the cLN of 3xTg mice. We also found differences in CD4+Foxp3+ Treg frequencies in the cLN (*SI Appendix*, Fig. S4 *E* and *F*). Taken together, we found induction of CD4+IL10+ in the spleen following 5 mo of treatment, where CD4+IL10+ Tregs would be expected to accumulate following prolonged treatment.

### Nasal Anti-CD3 Induces T Cell Migration to the Brain that Can Be Seen in Close Contact with Microglia.

**Fig. 3.**
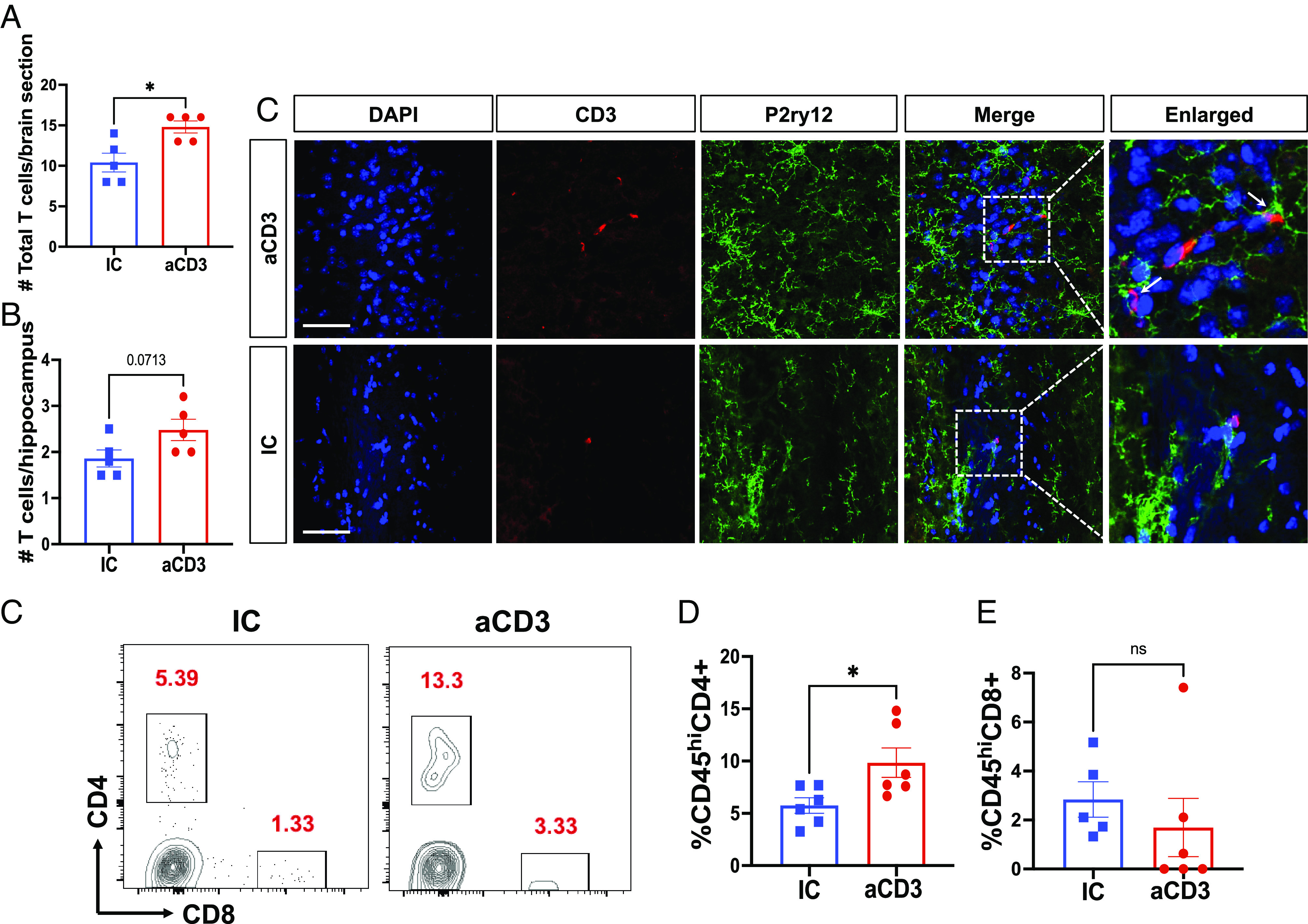
Nasal anti-CD3 promotes T cell migration to the brain where they associate with microglia. (*A* and *B*) Number of total CD3+ T cells in total brain (*A*) and in the hippocampus (*B*) of 3xTg female mice treated with 1 µg of either nasal anti-CD3 (aCD3) or isotype control (IC) 3×/week for 5 mo starting at 1 mo of age, measured by immunofluorescence (IF). n = 5 mice/group. Student’s *t* test. (*C*) Representative IF image of brain sections showing CD3+ T cells in close contact with microglial dendrites (white arrows). 20X, 50 µm. (*D* and *E*) Representative flow cytometry plots (*D*) and bar graphs (*E*) of treated mice in which CD4+ and CD8+ T cells were measured in total brain macerate. n = 5 to 6 mice/group (one outlier was removed from the CD8+ cell analysis). Student’s *t* test. Data are mean ± SEM. ns = not significant, *P < 0.05.

As shown above (Fig. 2), nasal anti-CD3 modulated the microglial transcriptome. We thus asked whether T cells from nasal anti-CD3-treated 3xTg mice could be found in the brain and whether they were in close contact with microglia. To address this, mice were treated with nasal anti-CD3, three times a week for 5 mo and brains were collected for IF staining, flow cytometry, and quantification of T cells. IF of the total brain (Fig. 3*A*) and to a lesser extent in the hippocampus (*P* = 0.0713) (Fig. 3*B*) showed increased numbers of T cells in the brains of nasal anti-CD3 treated animals vs. IC. As shown in Fig. 3*C*, we found CD3+ T cells in close contact with microglial dendrites (white arrows). We then performed flow cytometry to determine whether the infiltrating cells were predominantly CD4+ vs. CD8+ T cells and found increased frequencies of CD4+ cells in the brain but no changes in CD8+ T cells (Fig. 3 *D* and *E*). Thus, nasal anti-CD3 induces the migration of CD3+ T cells to the brain which then associate with microglia.

## Discussion

In addition to Aβ plaques and neurofibrillary tangles in the brain (2–4), neuroinflammation plays an important role in AD and has been identified as a major contributing factor to the neuronal death and cognitive impairment that occurs in the disease (6–9). The accumulation of abnormal protein aggregates in the brains of AD subjects triggers neuroinflammation and this cascade of events ultimately leads to neuronal dysfunction and death (1).

In the EAE MS model, we found that nasal anti-CD3 mAb dampened microglia and astrocyte activation by inducing IL-10-producing Tregs and ameliorated disease (22). Here, we demonstrate that nasal anti-CD3 also ameliorated disease in the 3xTg mouse model of AD. Treatment of animals for 5 mo with nasal anti-CD3 improved cognition and dampened microglial activation.

A striking finding of our study is that nasal anti-CD3 ameliorated cognition in 3xTg mice in an Aβ independent fashion as there were no changes in Aβ in either the cortex or hippocampus at 6 mo of age when cognitive deficits of 3xTg mice were improved. Our results are consistent with those of Dansokho et al., who reported that expansion of Tregs by low doses of IL-2 improved cognitive function in APP/PS1 mice without reducing amyloid plaque burden (17).

We presume that one of the mechanisms by which nasal anti-CD3 ameliorates disease in 3xTg mice relates to the expansion of IL10+ Tregs that migrate to the brain to dampen microglial activation and modulate the CNS environment. We found increased numbers of IL10-secreting T cells in the spleens of mice at 6 mo and in the brain, we found CD3+ T cells in close contact with microglial dendrites. Gene profiling of microglia following nasal anti-CD3 treatment demonstrated a switch from a MGnD or DAM phenotype to a M0 homeostatic phenotype. In addition, we found that nasal anti-CD3 altered the transcriptional landscape in the cortex and hippocampus of 3xTg mice in a sex-dependent fashion, though the mechanism underlying this effect is unknown. We believe that sex differences play an important role in the mechanism by which nasal anti-CD3 modulates gene expression in the hippocampus, cortex, and microglia, and this could explain the differences in short-term memory observed between males and females treated with nasal anti-CD3. It is important to note that the number of mice used for gene expression analysis (three to four per group) could have contributed to the lack of overlapping pathways in the cortex and hippocampus of male vs. female 3xTg mice treated with nasal anti-CD3.

An earlier study reported that a single intravenous administration of anti-CD3 mAb induced the upregulation of vascular cell adhesion molecule-1 (VCAM-1) on endothelial cells, favoring T cell adhesion to the endothelium and subsequent migration to the targeted organ (33). Although this mechanism could in part be involved in the nasal anti-CD3-induced T cell migration to the brain of 3xTg mice, we do not believe that this is the primary mechanism observed here as in our previous study we found localization of nasal anti-CD3 in the cLN and did not observe anti-CD3 in the blood or CNS following nasal administration (22).

An important question in considering immune therapy for AD relates to the timing of the intervention. It is generally accepted that anti-Aβ therapy is best given early in the disease, prior to it triggering phosphorylated tau, which is closely linked to cognition impairment. In terms of microglia, evidence suggests that there may be two stages of microglia activation in AD. Microglia are beneficial early in the disease and secrete antiinflammatory cytokines, whereas they are detrimental later in the disease at which time they secrete proinflammatory cytokines (34). We found that nasal anti-CD3 down-regulated several proinflammatory genes in microglia in 6-mo-old 3xTg mice, which suggests a beneficial role of nasal anti-CD3 via limiting microglial activation.

Antiamyloid therapy has been targeted to treat early AD. Few therapies are being developed for later stages of the disease. Given that nasal anti-CD3 decreases CNS inflammation and acts independent of effects on Aβ, it could be beneficial at later stages of disease when antiamyloid therapy is no longer effective. Interestingly, nasal anti-CD3 could be given in combination with antiamyloid therapy where it may amplify disease modification effects, and since nasal anti-CD3 decreases inflammation, it could theoretically find usefulness to treat the ARIA that is associated with antiamyloid therapy.

In summary, our findings suggest that nasal anti-CD3 has potential as a novel immunotherapy to treat AD that targets microglial cells. Foralumab, a fully human anti-CD3 mAb, has been successfully given to human subjects and has demonstrated immune effects with minimal toxicity (26). A pilot trial in subjects with COVID-19 (27, 28) and initial studies in subjects with progressive MS have shown positive effects. The results presented here provide the basis for testing nasal anti-CD3 in subjects with AD.

## Materials and Methods

### Animals.

All animal experiments were performed according to an Institutional Animal Care and Use Committee (IACUC)-approved protocol number 2016N000230. B6.Cg-Tg(APPSwe,tauP301L)1Lfa Psen1tm1Mpm/2J (3xTg) male and female, 1–6-mo-old mice were used in this study. Animals were purchased from the Jackson Laboratory (strain number 033930) and housed in a conventional specific pathogen-free facility at the Hale Building for Transformative Medicine, Brigham and Women’s Hospital, Harvard Medical School, and maintained on a 12-h light/dark cycle with food and water ad libitum.

### Behavioral Tests.

Behavioral experiments were carried out during daylight hours in a blinded fashion. Mice were allowed to acclimate for 3 to 4 wk prior to testing. On the day of testing, animals were acclimatized to the behavioral rooms for at least 30 min. Animals from different groups were tested consecutively. Data were digitally recorded using a camera-enabled Noldus EthoVision XT software. A) Y-maze. Mice were placed in a transparent Y-maze in which one of the arms was closed and were allowed to explore the two open arms for 3 min. Mice were placed back in their original cages for 10 min followed by another 3 min in the Y-maze but now with all three arms opened. Distance traveled (cm) in the familiar and novel arms was then quantified. B) Morris Water Maze. Spatial learning and memory were assessed using the Morris water maze test, as described in refs. 35 and 36. The test consisted of one visible trial, and four invisible trials followed by one probe trial. Trials were conducted on consecutive days. The pool was a circular white tank 122 cm in diameter, filled with water (23 ± 1 °C) to a depth of approximately 30 cm. Each mouse was allowed three attempts except for the probe trial during which each mouse was tested only once. For the visible trial on day 1, a transparent acrylic platform 10 cm in diameter was placed 1 cm above the water surface and was located approximately 15 cm from the edge of the maze. The platform location was changed with every visible trial. For the invisible trials on days 2, 3, 4, and 5, white paint was added, and the platform was submerged 1 cm below the surface of the water and kept in the same location throughout the four days. For the probe trial on day 6, the platform was completely removed. Visual clues of different colors and shapes were posted on the room walls. The water maze was divided into four equal quadrants, and a ceiling camera directly over the water maze was recording animal activity. Four start positions were designated along the perimeter of the tank, and the order in which these positions were used was changed each day. For each trial, the mouse was gently placed in the designated start position facing the wall. Mice were allowed 90 s to locate the platform using the spatial cues in the room, after which the animal remained on the platform for 15 s. Only during testing days 1, 2, and 3, if the mouse did not find the platform in the allotted 90 s, it was gently guided to the platform and allowed to remain there for 15 s and received the maximum score of 90 s for that trial. After each trial, mice were towel-dried and placed into a heated cage. Latency to platform and target quadrant and number of transitions to the platform were recorded.

### SMCxPRO Immunoassay for Measurement of Oligomeric Aβ (oAβ).

Immunoassay of oAβ was performed as described in ref. 37. 3xTg mice were treated with nasal anti-CD3, IC, or PBS for 11 mo starting at 1 mo of age and mice were then killed for oAβ measurement in brain extracts. Biotinylated capture mAbs (1C22) were conjugated to streptavidin magnetic particles (MPs) (Dynabeads MyOne, Thermo Fisher Scientific) at a ratio of 12.5 μg biotinylated antibody per milligram of MPs using a kit from Sigma Millipore. MPs with bound capture mAb were diluted to 50 μg/mL in the Aβ-Oligomer Assay Buffer (Tris buffer; 50 mM Tris, 150 mM NaCl, pH 7.6), with 1% Triton X-100, 0.0005% (w/v) d-des-thio-biotin and 0.1% bovine serum albumin), and 50μL of this suspension was added to 150 μL of the sample, standard, or blank and incubated at 600 rpm on a shaking incubator at 25 °C for 2 h. MPs were isolated using a magnet, and unbound material was removed by washing with 1× SMC wash buffer using a HydroFlex plate washer (Tecan Group AG). Fluorescently labeled (AlexaFluor-647) detection antibody 3D6 (20 μL, 200 ng/mL) was added to each well. MPs bearing the antibody–oligomer Aβ sandwich were then incubated with agitation using a Jitterbug shaker (Boekel) for 1 h at 25 °C. Unbound detection reagent was removed by washing (four times) with the wash buffer. The wash buffer was removed by aspiration, and the fluorescently labeled 3D6 detection antibody was released by shaking in an Elution Buffer B (11.5 μL/well) for 10 min at 25 °C. Then, 11 μL of the eluates were then transferred to the wells of a clean 96-well plate containing the Neutralization Buffer D (11 μL/well). The neutralized sample (20 μL/well) was then transferred to a black 384-well-read plate (Aurora) and read by the SMCxPRO instrument. The lower limit of reliable quantification (LLoQ) was defined as the lowest back interpolated standard that provides a signal two-fold the background with a percentage of recovery calculated between 80% and 100% and coefficient of variance (CV) ≤ 20%.

### Flow Cytometry.

Flow cytometry was performed as described in ref. 38. Briefly, spleen, cervical lymph nodes, and brains were removed upon completion of the experiments and cells were isolated for flow cytometric analyses. Dead cells were excluded based on 7-AAD (BD Biosciences) or the fixable viability dye Aqua Zombie (1:1,000; BioLegend) staining. For intracellular cytokine staining, cells were first stimulated for 3 h with PMA (phorbol 12-myristate 13-acetate; 50 ng/mL; Sigma-Aldrich) and ionomycin (1 μM; Sigma-Aldrich) and a protein-transport inhibitor containing monensin (1 μg/mL GolgiStop; BD Biosciences) before detection by staining with antibodies. Surface markers were stained for 25 min at 4 °C in Mg^2+^ and Ca^2+^ -free Hank's Balanced Salt Solution (HBSS) with 2% FCS, 0.4% Ethylenediaminetetraacetic acid (EDTA) (0.5 M), and 2.5% HEPES (1M) then were fixed in Cytoperm/Cytofix (eBioscience), permeabilized with Perm/Wash Buffer (eBiosciences). Flow-cytometric acquisition was performed on a Fortessa or Symphony A5 instruments (BD Biosciences) by using DIVA software (BD Biosciences) and data were analyzed with FlowJo software versions 10.1 (TreeStar Inc). Intracellular staining antibodies used are as follows: FITC-anti-Foxp3 (FJK-16s; 1:100; ThermoFisher), BV421-anti-IFN-γ (XMG1.2; 1:300; BioLegend), PE-Cy7-anti-IL-17A (eBio17B7; 1:300; eBioscience), and PE-anti-IL-10 (JES5.16E3; 1:100; eBioscience). Other antibodies included the following: FITC-anti-CD45 (30-F11; 1:200; BioLegend), AF700-anti-CD45 (30-F11; 1:200; BioLegend), BV605-anti-CD11b (M1/70; 1:300; BioLegend), AF700-anti-CD3e (17A2; 1:300; BioLegend), PECy7-anti-CD4 (GK1.5; 1:400; BioLegend), BUV496-anti-CD4 (GK1.5; 1:400; BD Biosciences), APC-anti-CD8b (YTS156.7.7; 1:300; BioLegend), Percp-Cy5.5-anti-Ly6C (HK1.4; 1:200; BioLegend), and microglia-specific 4D4 antibody described in our laboratory (31, 32).

### Nanostring.

Sorted microglia (CD45^int^CD11b^+^Ly6C^−^4D4^+^) or total cortex or hippocampus tissues gene expression was measured using the nCounter Mouse Myeloid (microglia) or Neuropathology (cortex and hippocampus) assays (NanoString Technologies). Differential testing was performed using the NSolver Advanced Analysis Module in which the distribution of each gene is used to select the optimal model for differential expression. Counts were normalized to a panel of housekeeping genes, and significant differences were detected by Student’s *t* test and statically relevant results consisted in *P* < 0.05. Statistically significant up- and down-regulated genes were separated, and R package clusterProfiler (v4.6.2)’s enrichGO function was used for pathway analysis. Pathways with a *P* value < 0.05 were kept, and the top 15 pathways were plotted, showing gene count and *P* value.

### Immunofluorescence Staining and Imaging.

For immunofluorescence staining, 20-µm brain sections encompassing the hippocampus and adjacent cortex were stained with the primary purified mouse antihuman anti-β-amyloid antibody (6E10; 1:300; BioLegend) followed by a donkey antimouse secondary antibody conjugated to Alexa Fluor-647 (1:200; Abcam) were used. For microglia and T cell staining, we used goat anti-P2ry12 (1:1,000, produced in house), Armenian hamster anti-CD3 mAb (145-2C11, 1:75, Abcam) followed by the secondary antibodies rabbit antigoat conjugated to Alexa Fluor 488 and rat anti-Armenia hamster conjugated to Cy2 (1:200, Jackson ImmunoResearch). Vectashield antifading mounting media with DAPI (Vector Laboratories) was used for nucleus staining. Images were acquired using a Leica DMi8 widefield fluorescence microscope or Zeiss LSM710 confocal microscope and analyzed using ImageJ.

### Statistics.

GraphPad Prism 9.0 was used for statistical analysis (unpaired, two-tailed Student’s *t* test or one-way ANOVA, followed by Tukey multiple comparisons). Statistical analysis for 16S rRNA sequencing data is described above. Differences were considered statistically significant with a *P* value of less than 0.05.

## Supplementary Material

Appendix 01 (PDF)

Dataset S01 (XLSX)

Dataset S02 (XLSX)

Dataset S03 (XLSX)

Dataset S04 (XLSX)

Dataset S05 (XLSX)

## Data Availability

All study data are included in the article and/or supporting information.

## References

[1] J. J. Manly , Estimating the prevalence of dementia and mild cognitive impairment in the US: The 2016 health and retirement study harmonized cognitive assessment protocol project. JAMA Neurol. **79**, 1242–1249 (2022). 36279130 10.1001/jamaneurol.2022.3543 PMC9593315

[2] C. Sato , Tau kinetics in neurons and the human central nervous system. Neuron **97**, 1284–1298.e1287 (2018). 29566794 10.1016/j.neuron.2018.02.015 PMC6137722

[3] B. J. Hanseeuw , Association of amyloid and tau with cognition in preclinical Alzheimer disease: A longitudinal study. JAMA Neurol. **76**, 915–924 (2019). 31157827 10.1001/jamaneurol.2019.1424 PMC6547132

[4] V. L. Villemagne , Amyloid beta deposition, neurodegeneration, and cognitive decline in sporadic Alzheimer’s disease: A prospective cohort study. Lancet Neurol. **12**, 357–367 (2013). 23477989 10.1016/S1474-4422(13)70044-9

[5] S. Thakur, R. Dhapola, P. Sarma, B. Medhi, D. H. Reddy, Neuroinflammation in Alzheimer’s disease: Current progress in molecular signaling and therapeutics. Inflammation **46**, 1–17 (2023). 35986874 10.1007/s10753-022-01721-1

[6] S. E. Hickman, E. K. Allison, J. El Khoury, Microglial dysfunction and defective beta-amyloid clearance pathways in aging Alzheimer’s disease mice. J. Neurosci. **28**, 8354–8360 (2008). 18701698 10.1523/JNEUROSCI.0616-08.2008 PMC2597474

[7] S. E. Hickman, J. El Khoury, Mechanisms of mononuclear phagocyte recruitment in Alzheimer’s disease. CNS Neurol. Disord. Drug. Targets **9**, 168–173 (2010). 20205643 10.2174/187152710791011982 PMC3684802

[8] R. M. Ransohoff, How neuroinflammation contributes to neurodegeneration. Science **353**, 777–783 (2016). 27540165 10.1126/science.aag2590

[9] K. S. Rawji , Immunosenescence of microglia and macrophages: Impact on the ageing central nervous system. Brain **139**, 653–661 (2016). 26912633 10.1093/brain/awv395 PMC5839598

[10] N. Habib , Disease-associated astrocytes in Alzheimer’s disease and aging. Nat. Neurosci. **23**, 701–706 (2020). 32341542 10.1038/s41593-020-0624-8 PMC9262034

[11] J. S. Sadick , Astrocytes and oligodendrocytes undergo subtype-specific transcriptional changes in Alzheimer’s disease. Neuron **110**, 1788–1805.e1710 (2022). 35381189 10.1016/j.neuron.2022.03.008 PMC9167747

[12] A. Griciuc, R. E. Tanzi, The role of innate immune genes in Alzheimer’s disease. Curr. Opin. Neurol. **34**, 228–236 (2021). 33560670 10.1097/WCO.0000000000000911 PMC7954128

[13] A. A. Pimenova, T. Raj, A. M. Goate, Untangling genetic risk for Alzheimer’s disease. Biol. Psychiatry **83**, 300–310 (2018). 28666525 10.1016/j.biopsych.2017.05.014 PMC5699970

[14] O. Butovsky , Identification of a unique TGF-beta-dependent molecular and functional signature in microglia. Nat. Neurosci. **17**, 131–143 (2014). 24316888 10.1038/nn.3599 PMC4066672

[15] H. Keren-Shaul , A unique microglia type associated with restricting development of Alzheimer’s disease. Cell **169**, 1276–1290.e1217 (2017). 28602351 10.1016/j.cell.2017.05.018

[16] S. Sakaguchi, T. Yamaguchi, T. Nomura, M. Ono, Regulatory T cells and immune tolerance. Cell **133**, 775–787 (2008). 18510923 10.1016/j.cell.2008.05.009

[17] C. Dansokho , Regulatory T cells delay disease progression in Alzheimer-like pathology. Brain **139**, 1237–1251 (2016). 26912648 10.1093/brain/awv408

[18] C. Toly-Ndour , MHC-independent genetic factors control the magnitude of CD4+ T cell responses to amyloid-beta peptide in mice through regulatory T cell-mediated inhibition. J. Immunol. **187**, 4492–4500 (2011). 21949026 10.4049/jimmunol.1003953

[19] H. Baek , Neuroprotective effects of CD4+CD25+Foxp3+ regulatory T cells in a 3xTg-AD Alzheimer’s disease model. Oncotarget **7**, 69347–69357 (2016). 27713140 10.18632/oncotarget.12469 PMC5342482

[20] A. Faridar , Restoring regulatory T-cell dysfunction in Alzheimer’s disease through ex vivo expansion. Brain Commun. **2**, fcaa112 (2020). 32954348 10.1093/braincomms/fcaa112 PMC7472911

[21] F. Ciccocioppo , The characterization of regulatory T-cell profiles in Alzheimer’s disease and multiple sclerosis. Sci. Rep. **9**, 8788 (2019). 31217537 10.1038/s41598-019-45433-3 PMC6584558

[22] L. Mayo , IL-10-dependent Tr1 cells attenuate astrocyte activation and ameliorate chronic central nervous system inflammation. Brain **139**, 1939–1957 (2016). 27246324 10.1093/brain/aww113 PMC4939696

[23] C. Kuhn , Mucosal administration of CD3-specific monoclonal antibody inhibits diabetes in NOD mice and in a preclinical mouse model transgenic for the CD3 epsilon chain. J. Autoimmun. **76**, 115–122 (2017). 27745778 10.1016/j.jaut.2016.10.001 PMC9815832

[24] H. Y. Wu, F. J. Quintana, H. L. Weiner, Nasal anti-CD3 antibody ameliorates lupus by inducing an IL-10-secreting CD4+ CD25- LAP+ regulatory T cell and is associated with down-regulation of IL-17+ CD4+ ICOS+ CXCR5+ follicular helper T cells J. Immunol. **181**, 6038–6050 (2008). 18941193 10.4049/jimmunol.181.9.6038 PMC2753458

[25] H. Y. Wu, R. Maron, A. M. Tukpah, H. L. Weiner, Mucosal anti-CD3 monoclonal antibody attenuates collagen-induced arthritis that is associated with induction of LAP+ regulatory T cells and is enhanced by administration of an emulsome-based Th2-skewing adjuvant J. Immunol. **185**, 3401–3407 (2010). 20720210 10.4049/jimmunol.1000836 PMC2962584

[26] T. Chitnis , Nasal administration of anti-CD3 monoclonal antibody modulates effector CD8+ T cell function and induces a regulatory response in T cells in human subjects. Front. Immunol. **13**, 956907 (2022). 36505477 10.3389/fimmu.2022.956907 PMC9727230

[27] T. G. Moreira , Nasal administration of anti-cd3 monoclonal antibody (foralumab) reduces lung inflammation and blood inflammatory biomarkers in mild to moderate COVID-19 patients: A pilot study. Front. Immunol. **12**, 709861 (2021). 34475873 10.3389/fimmu.2021.709861 PMC8406802

[28] T. G. Moreira , Nasal administration of anti-CD3 mAb (Foralumab) downregulates NKG7 and increases TGFB1 and GIMAP7 expression in T cells in subjects with COVID-19. Proc. Natl. Acad. Sci. U.S.A. **120**, e2220272120 (2023). 36881624 10.1073/pnas.2220272120 PMC10243127

[29] A. C. Stimmell , Impaired spatial reorientation in the 3xTg-AD mouse model of Alzheimer’s disease. Sci. Rep. **9**, 1311 (2019). 30718609 10.1038/s41598-018-37151-z PMC6361963

[30] S. Oddo , Triple-transgenic model of Alzheimer’s disease with plaques and tangles: Intracellular Abeta and synaptic dysfunction. Neuron **39**, 409–421 (2003). 12895417 10.1016/s0896-6273(03)00434-3

[31] S. Krasemann , The TREM2-APOE pathway drives the transcriptional phenotype of dysfunctional microglia in neurodegenerative diseases. Immunity **47**, 566–581.e569 (2017). 28930663 10.1016/j.immuni.2017.08.008 PMC5719893

[32] O. Butovsky , Modulating inflammatory monocytes with a unique microRNA gene signature ameliorates murine ALS. J. Clin. Invest. **122**, 3063–3087 (2012). 22863620 10.1172/JCI62636 PMC3428086

[33] S. D. Bergese, R. P. Pelletier, R. G. Ohye, D. A. Vallera, C. G. Orosz, Treatment of mice with anti-CD3 mAb induces endothelial vascular cell adhesion molecule-1 expression. Transplantation **57**, 711–717 (1994). 7511256 10.1097/00007890-199403150-00013

[34] Z. Fan, D. J. Brooks, A. Okello, P. Edison, An early and late peak in microglial activation in Alzheimer’s disease trajectory. Brain **140**, 792–803 (2017). 28122877 10.1093/brain/aww349 PMC5837520

[35] R. Morris, Developments of a water-maze procedure for studying spatial learning in the rat. J. Neurosci. Methods **11**, 47–60 (1984). 6471907 10.1016/0165-0270(84)90007-4

[36] H. Abou-El-Hassan , Vγ1 and Vγ4 gamma-delta T cells play opposing roles in the immunopathology of traumatic brain injury in males. Nat. Commun. **14**, 4286 (2023). 37463881 10.1038/s41467-023-39857-9 PMC10354011

[37] L. Liu , An ultra-sensitive immunoassay detects and quantifies soluble Abeta oligomers in human plasma. Alzheimers Dement **18**, 1186–1202 (2022). 34550630 10.1002/alz.12457 PMC8938295

[38] R. M. Rezende , Gamma-delta T cells modulate the microbiota and fecal micro-RNAs to maintain mucosal tolerance. Microbiome **11**, 32 (2023). 36814316 10.1186/s40168-023-01478-1 PMC9948450

