# Supplementary material for: Nasal administration of anti-CD3 monoclonal antibody ameliorates disease in a mouse model of Alzheimer’s disease
Source: Proc Natl Acad Sci U S A. 2023 Sep 5;120(37):e2309221120. doi: 10.1073/pnas.2309221120 (PMC10500187; doi:10.1073/pnas.2309221120)
Supplement: Supplementary file 1 — Appendix 01 (PDF) [file pnas.2309221120.sapp.pdf]

PNAS

Supporting Information for

Nasal administration of anti-CD3 monoclonal antibody ameliorates disease in a mouse model of Alzheimer's disease

Juliana R. Lopes[1,#,•], Xiaoming Zhang[1,#,•], Julia Mayrink[1], Bruna K. Tatematsu[1], Lydia Guo[1], Danielle S. LeServe[1], Hadi Abou-El-Hassan[1], Felipe Rong[1], Maria J. Dalton[1], Marilia G. Oliveira[1], Toby B. Lanser[1], Lei Liu[1], Oleg Butovsky[1], Rafael M. Rezende[1] and Howard L. Weiner[1,2*]

[1] Ann Romney Center for Neurologic Diseases, Department of Neurology, Brigham and Women's Hospital, Harvard Medical School. Boston, MA, 02115

[2] The Gene Lay Institute of Immunology and Inflammation, Brigham and Women's Hospital, Harvard Medical School. Boston, MA, 02115

[#] These authors contributed equally to this work.
[•] These authors share first authorship.
*Howard L. Weiner.

Email: hweiner@rics.bwh.harvard.edu

Author Contributions: J.R.L, X.Z. performed and analyzed the experiments. J.M., B.K.T, L.G., D.S.L., F.R., M.J.D. helped with mouse nasal treatment and immunofluorescence image acquisition and analysis. H.A. helped with behavioral tests. M.G.O. helped with flow cytometry experiments. T.B.L. performed pathway analysis. L.L. contributed with Aβ analysis. O.B. helped with microglial Nanostring analysis. R.M.R., H.L.W. designed and supervised the experiments and wrote the manuscript.

Competing Interest Statement: Authors declare no conflict of interest.

Classification: Biological Sciences, Immunology and Inflammation

Keywords: Nasal anti-CD3, 3xTg mice, microglia, T cells

This PDF file includes:
Supplementary Figures: 1 to 4

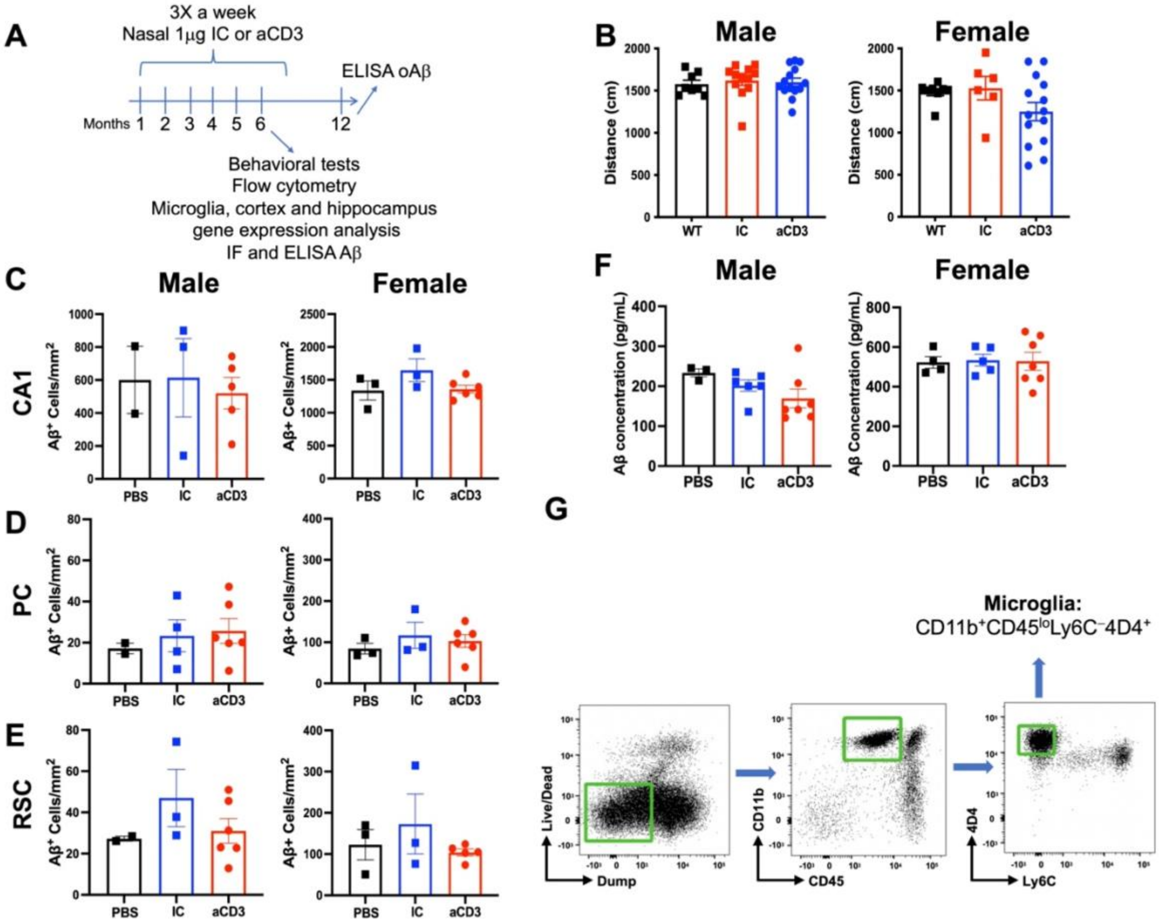

Supplementary Figure 1. Amyloid beta is not modulated by nasal anti-CD3. A) Scheme for nasal anti-CD3 mAb administration and experimental design. **B)** Distance traveled (cm) in the water maze probe day of healthy C57BL/6 WT mice and 3xTg mice treated with 1 μg of either nasal anti-CD3 (aCD3) or isotype control (IC) 3x/week for 5 months starting at 1 month of age. Male: n=8 WT, 12 IC, 14 aCD3. Female: n=8 WT, 6 IC, 14 aCD3. **C-E)** Intracellular Aβ quantification by immunofluorescence in the CA1 of the hippocampus **(B)**, prefrontal cortex (PC; **C**) and retrosplenial cortex (RSC; **D**) of male and female mice treated with 1 μg of either nasal anti-CD3 (aCD3) or isotype control (IC) 3x/week for 5 months starting at 1 month of age. n=2-5 mice/group. Analyses were performed using ImageJ. One way ANOVA with Tukey's posttest for multiple comparisons. **F)** ELISA of Aβ oligomers measured from brain extracts of 3xTg mice treated with 1 μg of nasal anti-CD3 (aCD3), isotype control (IC) or PBS 3x/week for 11 months starting at 1 month of age. n=3-7 mice/group. **G)** Gating strategy for microglia sorting. Data are mean ± s.e.m.

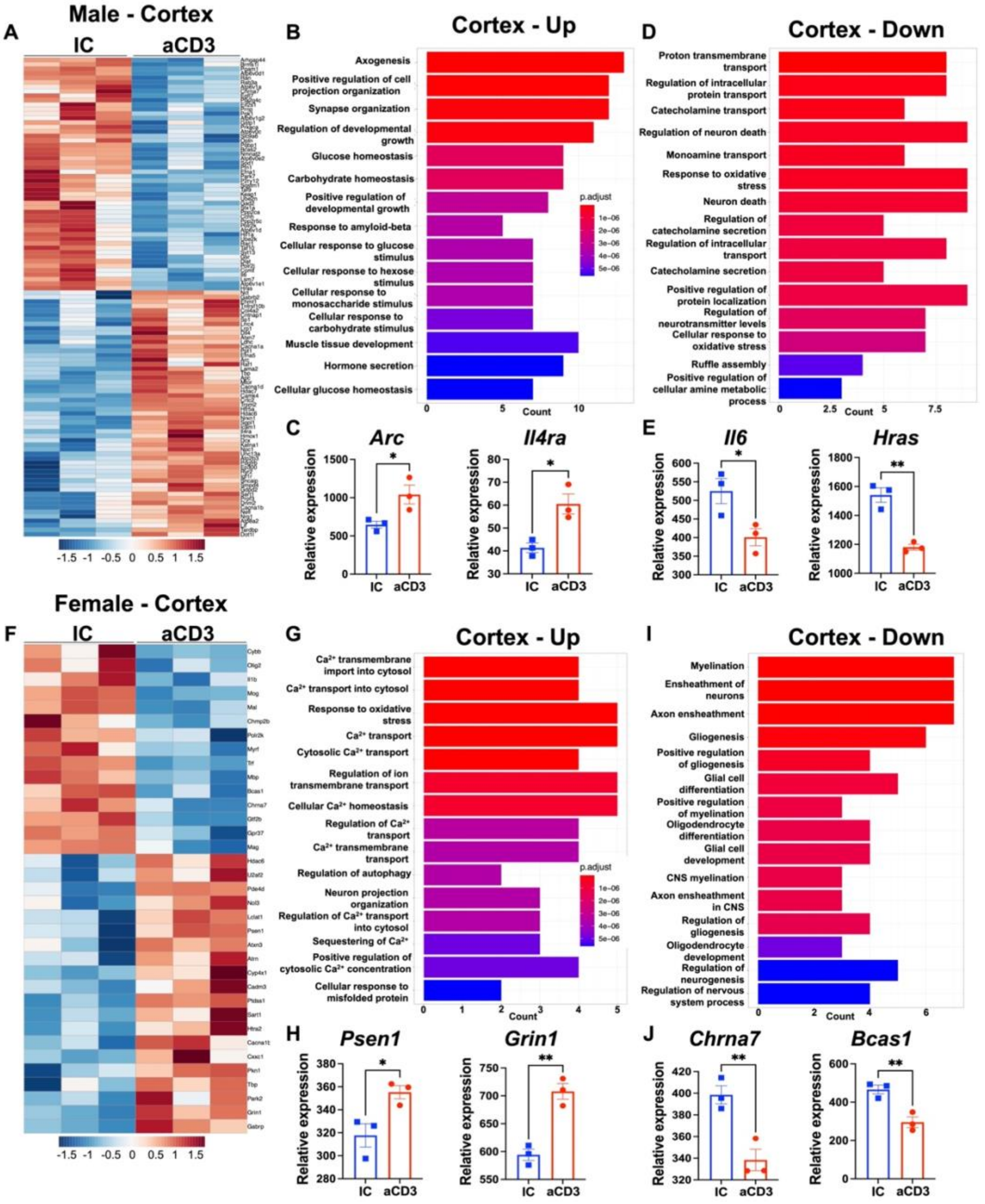

Supplementary Figure 2. Nasal anti-CD3 modulates the gene signature of the cortex. Whole cortical tissue was collected, and RNA extracted after the termination of the 5-month treatment of male and female mice with 1 μg of either nasal anti-CD3 (aCD3) or isotype control (IC) 3x/week starting at 1 month of age and gene expression analysis performed using the Nanostring mouse neuropathology panel. **A)** Heatmap of statistically significant genes in males ($p<0.05$). **B)** Pathway analysis of upregulated genes in aCD3 vs. IC treated males. **C)** Bar graph of representative upregulated genes referent to **(A)**. **D)** Pathway analysis of downregulated genes in aCD3 vs. IC treated males. **(E)** Bar graph of representative upregulated genes referent to **(A)**. **F)** Heatmap of statistically significant genes in females ($p<0.05$). **G)** Pathway analysis of upregulated genes in aCD3 vs. IC treated females. **H)** Bar graph of representative upregulated genes referent to **(F)**. **I)** Pathway analysis of downregulated genes in aCD3 vs. IC treated females. **(J)** Bar graph of representative downregulated genes referent to **(F)**. n=3 mice/group. Student's t-test. Data are mean ± s.e.m. ns=not significant, *$p<0.05$, **$p<0.01$.

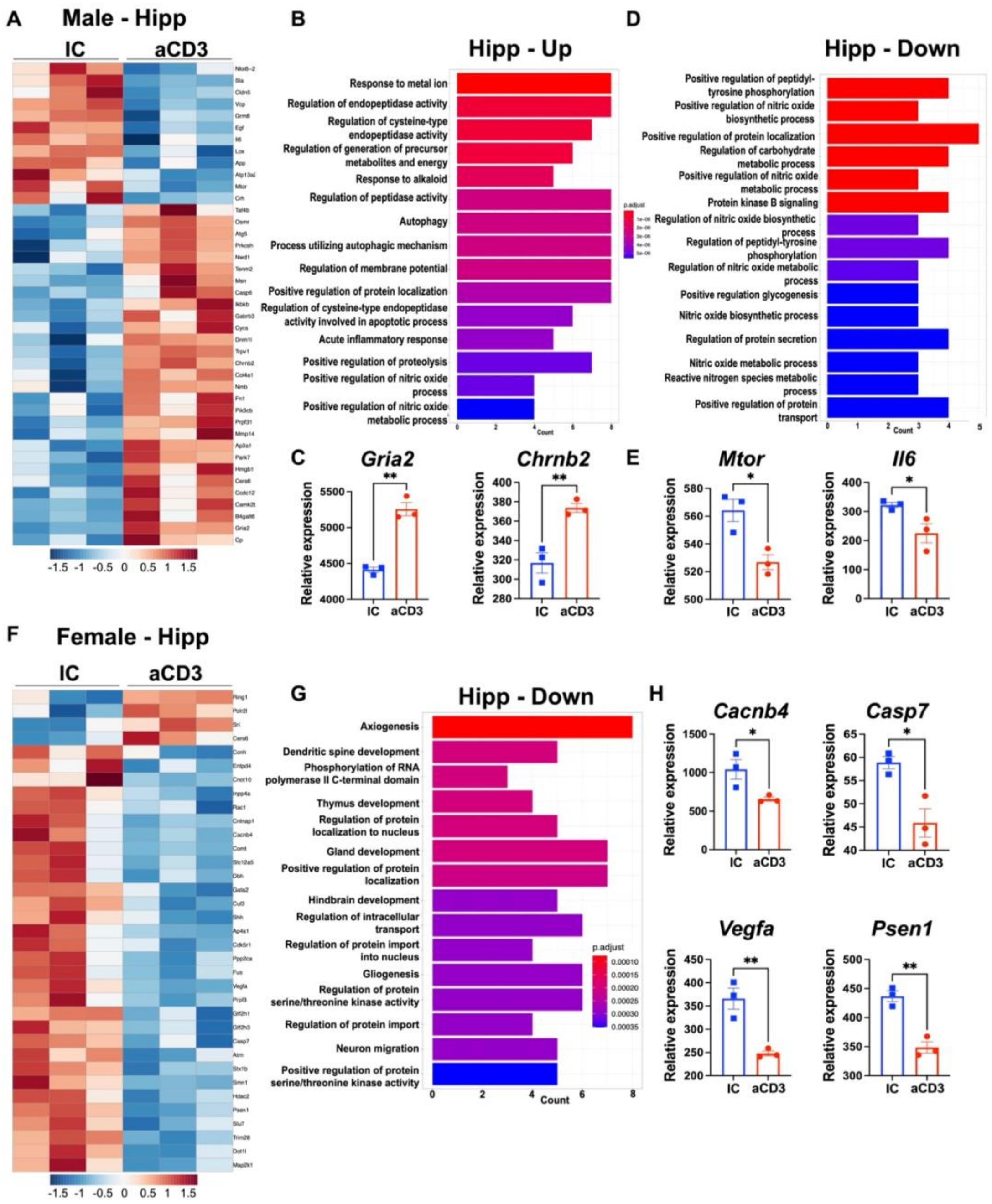

Supplementary Figure 3. Nasal anti-CD3 modulates the gene signature of the hippocampus. Whole hippocampal tissue was collected, and RNA extracted after the termination of the 5-month treatment of male and female mice with 1 μg of either nasal anti-CD3 (aCD3) or isotype control (IC) 3x/week starting at 1 month of age and gene expression analysis performed using the Nanostring mouse neuropathology panel. **A)** Heatmap of statistically significant genes in males ($p<0.05$). **B)** Pathway analysis of upregulated genes in aCD3 vs. IC treated males. **C)** Bar graph of representative upregulated genes referent to **(A)**. **D)** Pathway analysis of downregulated genes in aCD3 vs. IC treated males. **(E)** Bar graph of representative upregulated genes referent to **(A)**. **F)** Heatmap of statistically significant genes in females ($p<0.05$). **G)** Pathway analysis of downregulated genes in aCD3 vs. IC treated females. **(H)** Bar graph of representative downregulated genes referent to **(F)**. n=3 mice/group. Student's t-test. Data are mean $\pm$ s.e.m. *$p<0.05$, **$p<0.01$.

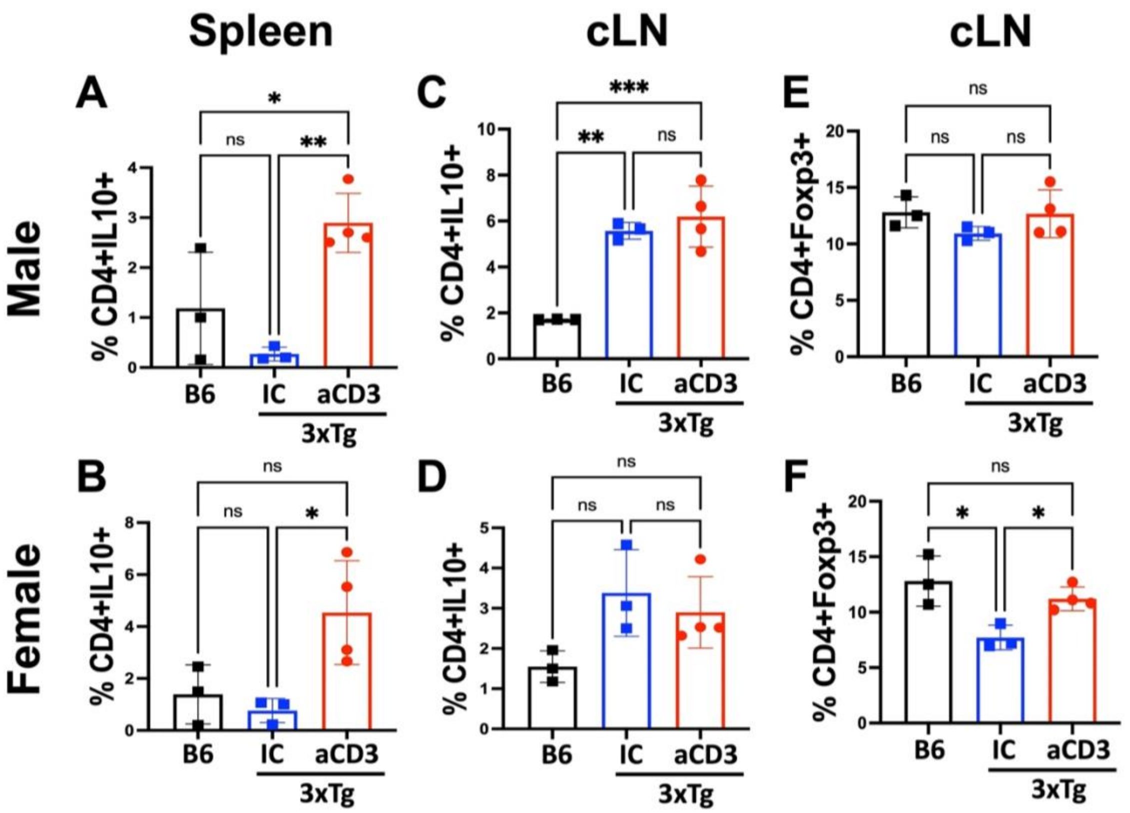

Supplementary Figure 4. Nasal anti-CD3 induces the expansion of Tregs. A-F) Bar graphs showing the percentage of CD4+IL10+ **(A-D)** and CD4+Foxp3+ **(E, F)** Tregs in the spleen and cervical lymph node (cLN) of male and female mice treated with 1 μg of either nasal anti-CD3 (aCD3) or isotype control (IC) 3x/week for 5 months starting at 1 month of age. Untreated C57BL/6 (B6) mice were used as a control for the disease. n=3-4 mice/group. One way ANOVA with Tukey's posttest for multiple comparisons. Data are mean ± s.e.m. ns=not significant, *p<0.05, **p<0.01, ***p<0.001.
